# Can theoretical values for chest wall compliance be used in ards patients?

**DOI:** 10.1186/2197-425X-3-S1-A999

**Published:** 2015-10-01

**Authors:** GQ Chen, M Xu, XL Chen, N Rittayamai, M Kim, I Soliman, Y-L Yang, J-X Zhou, L Brochard

**Affiliations:** Interdepartmental Division of Critical Care Medicine, University of Toronto, Toronto, Canada; Keenan Research Centre and Li Ka Shing Insitute, Department of Critical Care, St.Michael's Hospital, Toronto, Canada; Beijing Tiantan Hospital, Department of Critical Care Medicine, Capital Medical University, Beijing, China

## Introduction

To understand the impact of respiratory mechanics during mechanical ventilation, it is helpful to partition between the lungs and the chest wall. Esophageal pressure (Pes) is used to calculate chest wall compliance. However, esophageal pressure is not always used in the clinical arena. The value of chest wall compliance has been proposed to be estimated using 4% of the predicted value of vital capacity (VC) [1].

## Objectives

This study compared the difference between the predicted and the measured value of chest wall compliance in patients with ARDS.

## Methods

This observational study was conducted at St. Michael's Hospital in Toronto and Tiantan hospital in Beijing. Patients who met the Berlin definition of ARDS were eligible. Data recorded included age, height, gender, and SOFA score. Pes was measured using an esophageal balloon catheter (Cooper Surgical, United States) inflated with 1.0-ml air via the nose or mouth. Simultaneously, we measured other ventilator parameters that were used for chest wall compliance (Ccw-measured). We used an equation to calculate the predicted VC according to gender, age, and height [2]: 4% of the predicted VC was used as the value for predicted chest wall compliance (Ccw-predicted). We used the Bland-Altman [3] method to calculate the mean difference (bias) and the standard deviation of the differences (precision) between Ccw-predicted and Ccw-measured.

## Results

**Figure 1:**
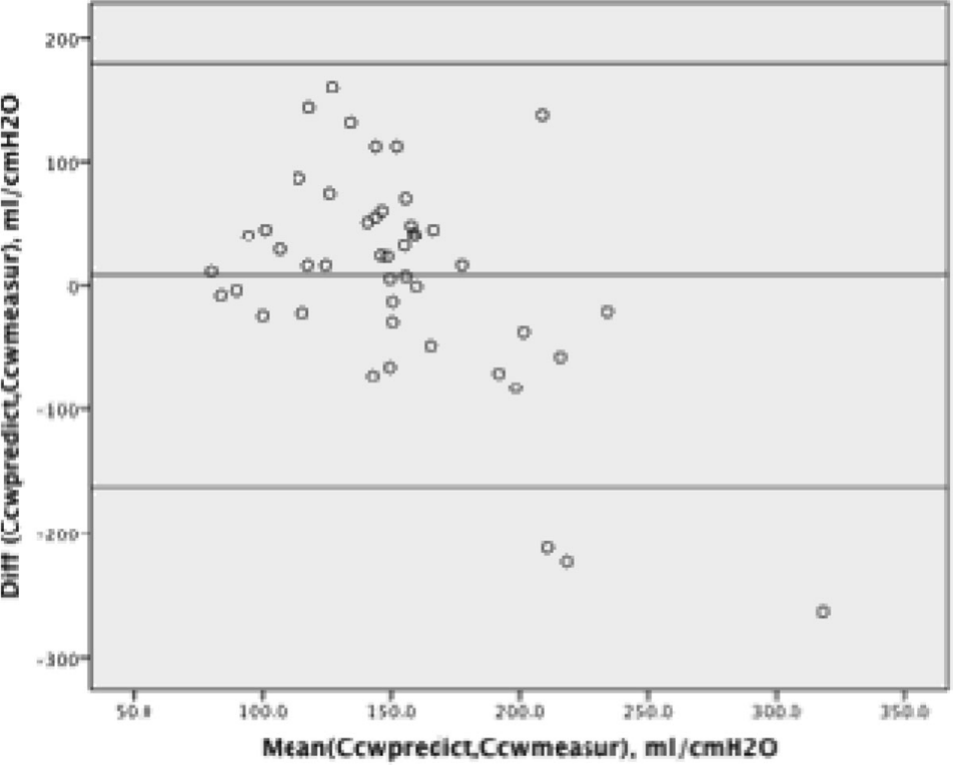
**Bland-Altman plot**.

A total of 46 patients were enrolled with the following characteristics: men/women: 31/15, age: 52 ± 22 years; 15% patients had mild ARDS, 63% patients had moderate ARDS, 22% patients had severe ARDS. They had no spontaneous breathing activity during the measurements. The mean values of the Ccw-predicted and Ccw-measured were 156 ± 41 ml/cmH2O and148 ± 78 ml/cmH2O (P = 0.52); the mean difference (bias) between Ccw-predicted and Ccw-measured was 8.4 ml/cmH2O. The standard deviation of the difference (precision) was 87.4 ml/cmH2O. Figure 1 is the Bland-Altman plot showing 95% limits of agreement as +180 ml/cmH2O and -163 ml/cmH2O.

## Conclusions

Although the average values of predicted and observed chest wall compliance are very close (small bias), the precision of the theoretical value is poor. The predicted value could be used as a first step approach but real measurements are needed to ascertain the influence of the chest wall.
